# Vacinação do Cardiopata contra COVID-19: As Razões da Prioridade

**DOI:** 10.36660/abc.20210012

**Published:** 2021-02-19

**Authors:** Wolney de Andrade Martins, Gláucia Maria Moraes de Oliveira, Andréa Araujo Brandão, Ricardo Mourilhe-Rocha, Evandro Tinoco Mesquita, José Francisco Kerr Saraiva, Fernando Bacal, Marcelo Antônio Cartaxo Queiroga Lopes

**Affiliations:** 1 Universidade Federal Fluminense NiteróiRJ Brasil Universidade Federal Fluminense (UFF), Niterói, RJ - Brasil; 2 Sociedade de Cardiologia do Estado do Rio de Janeiro Rio de JaneiroRJ Brasil Sociedade de Cardiologia do Estado do Rio de Janeiro (SOCERJ),Rio de Janeiro, RJ - Brasil; 3 Complexo Hospitalar de Niterói NiteróiRJ Brasil Complexo Hospitalar de Niterói (CHN), Niterói, RJ - Brasil; 4 Universidade Federal do Rio de Janeiro Rio de JaneiroRJ Brasil Universidade Federal do Rio de Janeiro (UFRJ), Rio de Janeiro, RJ - Brasil; 5 Sociedade Brasileira de Cardiologia Rio de JaneiroRJ Brasil Sociedade Brasileira de Cardiologia (SBC), Rio de Janeiro, RJ - Brasil; 6 Universidade do Estado do Rio de Janeiro Rio de JaneiroRJ Brasil Universidade do Estado do Rio de Janeiro (UERJ), Rio de Janeiro, RJ - Brasil; 7 Hospital Pró-Cardíaco Rio de JaneiroRJ Brasil Hospital Pró-Cardíaco, Rio de Janeiro, RJ - Brasil; 8 Pontifícia Universidade Católica de Campinas CampinasSP Brasil Pontifícia Universidade Católica de Campinas (PUC), Campinas, SP - Brasil; 9 Universidade de São Paulo São Paulo Brasil Universidade de São Paulo (USP), São Paulo – Brasil

**Keywords:** Infecções por Coronavírus, COVID-19, Betacoronavírus, Pandemia, Vacinação, Vacinas, Doenças Cardiovasculares, Influenza Humana, Política de Saúde

## A pandemia da COVID-19

A Organização Mundial da Saúde reconheceu a COVID-19 como pandemia em 11 de março de 2020 e, desde então, essa emergência de saúde pública converteu-se na principal causa de óbitos no mundo, o que tornou seu enfrentamento uma prioridade inquestionável. Ao escrevermos este editorial contabilizávamos 86.969.386 casos confirmados e 1.915.657 mortes por COVID-19 no mundo, dos quais 8.013.708 casos ocorreram no Brasil e resultaram em 201.460 mortes.^1^ Segundo as projeções do Institute for Health Metrics and Evaluation (IHME)^2^ o Brasil atingirá 248.476 mortes por COVID-19 em 4 de abril de 2021. As projeções para o dia 19 de março de 2021 estimam 242.738 [232.202 – 255.044] mortes, que poderiam ser reduzidas para 241.668 [231.337 – 253.770], caso a vacina fosse rapidamente administrada, e para 223.910 [215.565 – 233.360], caso a máscara fosse utilizada em 95% das situações em todos os locais. Preocupa a magnitude do número de casos e óbitos por única doença em tão pouco tempo. No momento, quando há crescimento de novos casos e internações, começar a vacinar terá impacto na redução de mortes e internações em intervalo curto. Apesar do esforço da comunidade científica, não há um tratamento específico para bloquear a replicação viral. Nesse sentido, programas de vacinação são poderosos aliados e, em virtude do notável progresso da ciência, já dispomos desse recurso.

O Brasil, por intermédio do Sistema Único de Saúde (SUS), tem se notabilizado pelo êxito da execução de programas de vacinação da sua população. É premente a instituição de política pública para vacinar dentro dos princípios do SUS: universalidade, integralidade e equidade. Entretanto, diante dos movimentos antivacina que têm surgido em nível mundial, é necessário um forte esforço para que se obtenha a adesão da população. No passado, já enfrentamos com muito êxito essa incredulidade, como na Revolta da Vacina vivida por Oswaldo Cruz. Miremo-nos nesse exemplo para superar essa grave crise sanitária.

## A epidemiologia das doenças cardiovasculares na COVID-19

No Brasil, entre 17 de março e 22 de maio de 2020, houve número maior de óbitos nas capitais das regiões Norte, Nordeste e Sudeste, especialmente em São Paulo, Rio de Janeiro, Fortaleza, Recife, Belém e Manaus, com menor ocorrência de notificação de óbitos nas capitais do Sul e do Centro-Oeste e nos municípios do interior. Observamos aumento de notificação de óbitos por causas cardiovasculares inespecíficas em todas as regiões, nas capitais e no interior, principalmente nas regiões Norte, Nordeste e Sudeste. Por outro lado, houve redução percentual das notificações de óbitos por síndrome coronariana aguda (SCA) e acidente vascular cerebral (AVC), com maior magnitude no Nordeste, seguindo-se as regiões Centro-Oeste e Sudeste (capital e interior).^3^

A pandemia pelo coronavírus em 2020 no Brasil aumentou o número de óbitos gerais, por doenças cardiovasculares (DCV) e por causas inespecíficas, assim como o número de mortes súbitas em domicílio. As diferenças regionais exprimem as desigualdades socioeconômicas e étnicas de um país continental, sendo ainda consequência de um sistema de saúde com recursos heterogêneos e mal distribuídos.^3^

COVID-19 é a novidade pandêmica. A DCV é nossa realidade endêmica, consolidada e irresoluta. Ambas comprometem a saúde em todos os aspectos, individuais e coletivos, físicos, psíquicos, sociais e econômicos. Em comum, ceifam vidas produtivas e promissoras.

Ainda carecemos de estudos duplo-cegos, randomizados, placebo-controlados que mostrem a relação de causalidade entre vacinação contra COVID-19 e benefício nos cardiopatas. Utilizemos então a melhor evidência disponível.

## As vacinas e o impacto na humanidade

Apesar de terem surgido antes mesmo dos imunologistas, as vacinas provocaram impacto no controle ou até erradicação de doenças outrora devastadoras. A varíola matava 29% das crianças na Londres dos séculos XVII e XVIII e foi declarada extinta em 1980. Quem entre nós fez diagnóstico de miocardite diftérica nos últimos 10 anos? Quantos casos de tétano neonatal foram internados em seu hospital em 2020? Vacinações mudaram a história natural das epidemias de difteria em 1940, de poliomielite em 1956, de coqueluche em 1950, de sarampo em 1968, de doença meningocócica em 1999, entre muitas outras. Entretanto, o vacilo nas campanhas resultou, invariavelmente, em reincidência.^4^

## O modelo ‘influenza’

A vacinação contra influenza é a experiência exitosa baseada em evidências mais próxima da atual situação pandêmica pela COVID-19. Apesar de a vacinação contra influenza ser recomendada pelas principais diretrizes em cardiologia, a cobertura vacinal é baixa e aumentou pouco na última década.^5^ A vacinação depende, em muito, da recomendação do cardiologista, que é, sobretudo, “o clínico” do cardiopata, ouvido em diversas situações. O conhecimento e consequente convencimento sobre a necessidade da vacina é determinante para sua difusão. A vacina da influenza é o exemplo inequívoco: acha-se disponível, é de fácil acesso em campanhas, mas sua cobertura não ultrapassa 25% dos pacientes com insuficiência cardíaca (IC).^5 , 6^

A necessidade da vacinação contra influenza em cardiopatas foi determinada primeiramente pelos relatos históricos de aumento de mortalidade nas epidemias e, posteriormente, por estudos epidemiológicos.^5^ O Quadro 1 apresenta evidências que embasaram tais recomendações.^7 - 15^ Hoje, sabe-se que a vacinação é medida eficaz na prevenção secundária, pois reduz internações hospitalares por IC, AVC e SCA, além de reduzir mortalidade geral de modo mais expressivo que muitos medicamentos ou intervenções.^5 , 6^

**Quadro 1:** – Principais evidências que embasaram a recomendação da vacinação contra influenza em cardiopatas

Autor	Ano	n	Principais conclusões
Nichol KL et al.^7^	2003	286.383 idosos	Vacina contra influenza reduziu mortalidade geral em 48%, hospitalizações por doença cardíaca em 19% e AVC entre 16% e 23%
Yap FHY et al.^8^	2004	17.226 internações por DCNT	Influenza causou excedente de 45,6% de internações por IC
Sandoval C et al.^9^	2008	5.448 pacientes com disfunção ventricular sistólica	O risco de hospitalização por IC é 8% a 10% maior durante a estação de influenza, independentemente de como é definida
Jorge JEL et al.^10^	2009	6.596 hospitalizações por IC	A sazonalidade com maior número de internações por IC descompensada ocorre também em regiões de clima tropical
Estabragh ZR & Mamas MA^11^	2013	40 estudos	Influenza leva a efeito direto: miocardite com choque cardiogênico, aumento de IAM, diminuição da mortalidade cardiovascular após vacinação
Wu WC et al.^12^	2014	107.045 pacientes com IC	Vacinação contra influenza reduziu mortalidade de pacientes com IC em 30 dias e 1 ano
Caldeira D et al.^13^	2015	4 estudos	Vacinação contra influenza é eficaz na prevenção secundária em pacientes com doença cardiovascular. Faltam dados para comprovar a mesma ação em prevenção primária
Blaya-Nováková V et al.^14^	2016	227.984 pacientes seguidos por 5 anos	Vacinação contra influenza reduziu risco de mortalidade global no inverno em 41% por ano
Fang YA et al.^15^	2016	4.406 pacientes com IRC e idade ≥55 anos.	Idosos com doença renal crônica que receberam vacinação anual contra influenza apresentam menor risco de hospitalizações por IC

*AVC: acidente vascular cerebral; DCNT: doenças crônicas não transmissíveis; IC: insuficiência cardíaca; IAM: infarto agudo do miocárdio; IRC: insuficiência renal crônica.*

## As infecções e a síndrome inflamatória sistêmica

**Figura 1:**
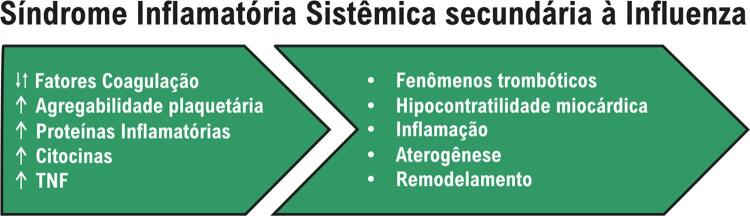
– Fisiopatologia das alterações cardiovasculares secundárias à inflamação sistêmica na influenza.

A influenza predispõe a pneumonia bacteriana secundária e, dessa forma, descompensa o paciente com IC. Sim, é fato. Entretanto, deve-se ressaltar que a síndrome inflamatória sistêmica secundária à influenza leva a alteração dos fatores de coagulação, hiperagregabilidade plaquetária, além de aumento das proteínas de fase inflamatória, das citocinas e do fator de necrose tumoral. Consequentemente, tem-se incremento dos fenômenos trombóticos e depósito de fibrina, hipocontratilidade do cardiomiócito, inflamação e aceleração da aterogênese e do remodelamento ( Figura 1 ). Assim, explica-se facilmente o porquê da redução de SCA e AVC nos pacientes vacinados em relação aos controles nos ensaios clínicos e observações epidemiológicas.^5 , 16^

A COVID-19 trouxe à tona a discussão dos mesmos mecanismos e manifestações já muito bem estudados na influenza. É inegável que a resposta inflamatória apresentada na COVID-19 seja mais exuberante e grave, associada ao risco de trombose. Portanto, conhecemos as peculiaridades da imunização nesse subgrupo de indivíduos e somos capazes de recomendar providências eficientes para ampliar as chances de sucesso do programa de imunização contra a COVID-19.

## COVID-19 e grupos de risco

Desde as primeiras séries publicadas a partir da China e da Itália, a gravidade da COVID-19 sobressaiu nos portadores das doenças crônicas não transmissíveis, muito provavelmente tomados em comum pela inflamação sistêmica crônica.^17^ Descontadas as confusões suscitadas por interpretações inadequadas de estudos ecológicos, o conceito de grupo de risco manteve-se nas publicações subsequentes. Na verdade, fato já conhecido desde os estudos da influenza. O paciente com IC é um indubitável exemplo de grupo prioritário e a Sociedade Brasileira de Cardiologia (SBC) já se manifestou a respeito.^18^

Recentemente, a SBC foi convidada pelo Ministério da Saúde para integrar a Câmara Técnica e revisar o Programa Nacional de Imunização contra a COVID-19 e apontou sugestões relativas à vacinação em enfermos acometidos por todas as DCV, definindo e especificando grupos prioritários para a vacinação ( Quadro 2 ).

**Quadro 2:** – Doenças cardiovasculares e cerebrovasculares prioritárias para vacinação contra COVID-19. Sugestões oferecidas pela Sociedade Brasileira de Cardiologia ao Programa Nacional de Imunizações do Ministério da Saúde

Síndrome/Doença cardiovascular ou cerebrovascular	Definição
Insuficiência cardíaca	IC com fração de ejeção reduzida, intermediária ou preservada, em estágios B, C ou D, independentemente de classe funcional da *New York Heart Association* Pós-transplante cardíaco (usar vacinas de vírus inativado)
Cor-pulmonale e hipertensão pulmonar	Cor-pulmonale crônico, hipertensão pulmonar primária ou secundária
Hipertensão arterial resistente	Quando a PA permanece acima das metas recomendadas com o uso de três ou mais anti-hipertensivos de diferentes classes, em doses máximas preconizadas e toleradas, administradas com frequência, dosagem apropriada e comprovada adesão ou PA controlada em uso de quatro ou mais fármacos anti-hipertensivos
Hipertensão arterial estágio 3	PA sistólica ≥180 mmHg e/ou diastólica ≥110 mmHg independentemente da presença de LOA ou comorbidade
Hipertensão arterial estágios 1 e 2 com LOA e/ ou comorbidade	PA sistólica entre 140 e 179 mmHg e/ou diastólica entre 90 e 109 mmHg na presença de LOA e/ ou comorbidade
Cardiopatia hipertensiva	Cardiopatia hipertensiva (hipertrofia ventricular esquerda ou dilatação, sobrecarga atrial e ventricular, disfunção diastólica e/ou sistólica, lesões em outros órgãos-alvo)
Síndromes coronarianas	Síndromes coronarianas crônicas ( *angina pectoris* estável, cardiopatia isquêmica, pós infarto agudo do miocárdio, outras)
Valvopatias	Lesões valvares com repercussão hemodinâmica ou sintomática ou com comprometimento miocárdico (estenose ou insuficiência aórtica, estenose ou insuficiência mitral, estenose ou insuficiência pulmonar, estenose ou insuficiência tricúspide, outras)
Miocardiopatias e pericardiopatias	Miocardiopatias de qualquer etiologia ou fenótipo Pericardite crônica Cardiopatia reumática
Doenças da aorta, dos grandes vasos e fístulas arteriovenosas	Aneurismas, dissecções, hematomas da aorta e demais grandes vasos
Arritmias cardíacas	Arritmias cardíacas com importância clínica e/ou cardiopatia associada (fibrilação e *flutter * atriais, outras)
Cardiopatias congênitas no adulto	Cardiopatias congênitas com repercussão hemodinâmica, crises hipoxêmica, insuficiência cardíaca, arritmias, comprometimento miocárdico.
Próteses valvares e dispositivos cardíacos implantados	Portadores de próteses valvares biológicas ou mecânicas e dispositivos cardíacos implantados (marca-passos, cardiodesfibriladores, ressincronizadores, assistência circulatória de média e longa permanência)
Doença cerebrovascular	Acidente vascular cerebral isquêmico ou hemorrágico, ataque isquêmico transitório, demência vascular

*IC: insuficiência cardíaca; PA: pressão arterial; LOA: lesão de órgão-alvo. Fonte: correspondência enviada ao Programa Nacional de Imunizações do Ministério da Saúde, em 02/01/2021.*

## As atuais perspectivas com as diferentes vacinas contra COVID-19

Ainda há poucas vacinas testadas em estudos fase 2 ou 3. No entanto, os resultados são muito positivos e impactantes, tanto em segurança quanto em eficácia. Merece destaque que as vacinas apoiadas pela Pfizer,^19^ Moderna^20^ e AstraZeneca^21^ incluíram idosos, cardiopatas, diabéticos, obesos graves, afrodescendentes e latinos. E, apesar do número relativamente reduzido, essa inclusão permite-nos inferir a segurança e a eficácia em pacientes cardiopatas. Os efeitos adversos observados foram locais, porém menos comuns nos mais idosos. Os efeitos cardiovasculares observados, como hipertensão, bradicardia, taquicardia, fibrilação atrial, SCA ou tromboembolia pulmonar, tiveram frequência menor que 0,1% e foram semelhantes entre os vacinados e os que receberam placebo ( Quadro 3 ).

**Quadro 3:** – Características demográficas e clínicas dos voluntários vacinados contra COVID-19 nos ensaios clínicos

Características	BNT162b2 (Pfizer/BioNTech)^19^	mRNA-1273 (Moderna / NIAID / NIH)^20^	ChAdOx1 COV 003 (Oxford/ AstraZeneca)^21^
Número de voluntários (n)	44.820	30.420	23.848 (4.088 Brasil)
Faixa etária (anos)	16 a 91	18 a 95	≥18
Mediana idade (anos)	52 (42,2% ≥55)	51 (24,8% ≥65)	-
Afrodescendentes (%)	9	10,2	10,4**
Latinos ou Hispânicos (%)	28	24,8	-
Efeitos adversos	Dor local foi mais comum em vacinados Mais frequente nos mais jovens	Dor local após injeção mais frequente no grupo vacinado que placebo Os efeitos adversos são mais comuns após a segunda dose Mais comuns nos mais jovens	Indisponível
Efeitos cardiovasculares	Indisponível	Bradicardia, síncope, taquicardia, síndrome coronariana aguda, fibrilação atrial, hipertensão, hipotensão, todos <0,1% e semelhante frequência entre placebo e vacinados	Indisponível
Eficácia (%)	95,0 [IC 90,3 – 97,6]	94,1 [IC 89,3 – 96,8]	64,2 [IC 30,7 – 81,5]
Eficácia em subgrupos	Semelhante nos subgrupos, incluindo hipertensos, idosos, obesos e brasileiros	Semelhante em subgrupo de risco para COVID-19 grave	Indisponível
Obesos (IMC>30 kg/m^2^) (%)	35,1	6,7	20,4
Diabéticos (%)	38,4*	9,5	3,0**
Doença cardiovascular (%)	2,7*	4,5	12,0**

IMC: índice de massa corpórea. (*) Cálculo aproximado a partir de dados publicados em apêndices do trabalho. (**) Dados referem-se à casuística do trabalho realizado no Brasil (COV 003). Nota: dados obtidos e analisados com critérios diferentes, portanto, com limitação quanto à comparação.

É oportuno destacar que o Brasil firmou parcerias, desde maio de 2020, para pesquisa e desenvolvimento de vacinas que incluem transferência de tecnologia por intermédio da Fundação Oswaldo Cruz e do Instituto Butantan. A vacina desenvolvida pela AstraZeneca e pela Universidade de Oxford já teve seus resultados preliminares publicados e encontra-se em uso na Inglaterra. Essa vacina será produzida em larga escala entre nós. Concomitantemente, a vacina denominada CoronaVac, desenvolvida pelo laboratório Sinovac, será produzida no Instituto Butantan. Esse Instituto informou nos meios de comunicação que “em estudo clínico com 12.400 voluntários, o imunizante demonstrou eficácia de 78% para casos leves e de 100% para casos moderados e graves.”^22^ Portanto, há perspectivas objetivas de dispormos de vacinas.

É necessário enfatizar que o Brasil tem uma das mais avançadas legislações sanitárias do mundo. A Constituição Federal consagra o acesso à saúde como direito fundamental: “ *A saúde é um direito de todos e dever do Estado, garantido mediante políticas sociais e econômicas ...”.* Assim, políticas públicas de saúde seguras, eficazes, efetivas e custo-efetivas fazem parte do mínimo existencial de cada brasileiro, devendo ser ofertadas de maneira universal, integral e gratuita. Enquadram-se nesse contexto as campanhas de vacinação, verdadeiro patrimônio consolidado dos brasileiros e orgulho nacional. À vista disso, criar todas as condições para ofertar vacinas em um amplo programa de imunização contra a COVID-19 é “ *direito de todos e dever do Estado* ”, sob pena do dever constitucional converter-se em promessa inconsequente, frustrando as justas expectativas depositadas no Estado brasileiro.

## Por que vacinar?

**Figura 2:**
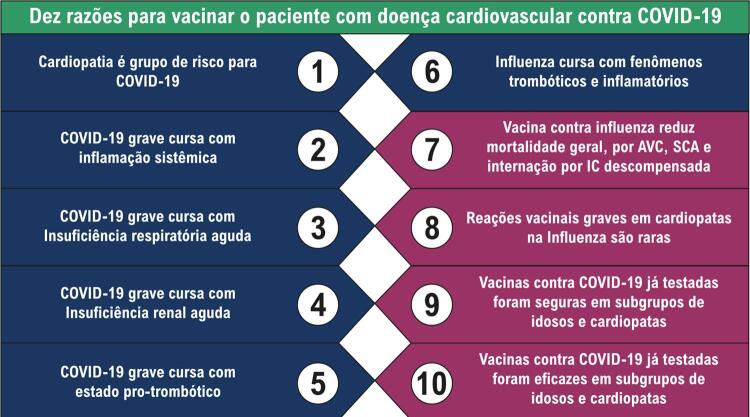
– Dez razões para vacinar o paciente com doença cardiovascular contra COVID-19. AVC: acidente vascular cerebral; SCA: síndrome coronariana aguda; IC: insuficiência cardíaca.

Resumimos as dez razões para indicar a vacina ao seu paciente na Figura 2 . É nosso ponto de vista, baseado na melhor evidência existente, que devemos nos engajar na difusão desse conhecimento e motivar nossos pacientes. No entanto, impõe-se a manutenção das eficazes e comprovadas medidas de prevenção ao contágio pela COVID-19: higienização das mãos, uso de máscaras e distanciamento social. Em que pese que o programa de vacinação poderá contribuir para a minimização do contágio, certamente as medidas clássicas de prevenção deverão ser mantidas até que se prove definitivamente o benefício do programa de vacinação.

## A Sociedade Brasileira de Cardiologia e seu compromisso com a ciência

A SBC não fugirá ao legado histórico, edificado no exemplo de Carlos Chagas, Dante Pazzanese e nossos pioneiros, transmitido por mais de sete décadas aos mais de 14.000 associados, confirmado em seu propósito social. É objetivo da SBC “ *Expandir, divulgar e incentivar, em todos os níveis, o conhecimento, o diagnóstico, a prevenção e o tratamento das DCV, desenvolvendo campanhas educativas em conjunto com o poder público e com outras entidades e associações, e divulgar, junto à sociedade civil, os aspectos epidemiológicos das DCV, esclarecendo-a quanto às possibilidades de prevenção e tratamento* ”.^23^

Apesar do elevado custo em vidas perdidas, a busca por solução eficiente para a pandemia, trouxe-nos rápido avanço nas pesquisas, alicerçado em ciência de boa qualidade, deixando um notável legado e conquistas. No curso de um ano, descreveram-se o quadro clínico, o perfil epidemiológico e o agente etiológico em nível molecular, aprimoraram-se cuidados, refutaram-se tratamentos empíricos e fúteis e produziram-se vacinas testadas em ensaios clínicos. É a ciência em sua fascinante evolução por eficácia em prol da qualidade e quantidade de vida. Mas, a grande lição tem sido a necessidade do fortalecimento do sistema de saúde, o nosso SUS. A defesa intransigente do SUS, em síntese, é a defesa da dignidade da pessoa humana, compromisso fundamental do Estado brasileiro. A SBC e as demais sociedades científicas devem se aliar na luta pelo progresso e difusão da ciência e pela consecução de políticas públicas capazes de melhorar a vida de cada um dos mais de 220 milhões de brasileiros. Os princípios que nortearam a criação da SBC em 1943, no meio da Segunda Guerra Mundial, são os mesmos que nos motivam nesta crise sanitária sem precedentes.
