# Supplementary material for: Vacinação do Cardiopata contra COVID-19: As Razões da Prioridade
Source: Arq Bras Cardiol. 2021 Feb 19;116(2):213–8. [Article in Portuguese] doi: 10.36660/abc.20210012 (PMC7909963; doi:10.36660/abc.20210012)
Supplement: Translation

# Translation: Vaccinating Patients with Heart Disease Against COVID-19: The Reasons for Priority

**Authors:** Wolney de Andrade Martins, Gláucia Maria Moraes de Oliveira, Andréa Araujo Brandão, Ricardo Mourilhe-Rocha, Evandro Tinoco Mesquita, José Francisco Kerr Saraiva, Fernando Bacal, Marcelo Antônio Cartaxo Queiroga Lopes

**Affiliations:** 1 Brazil Universidade Federal Fluminense (UFF), Niterói, RJ - Brazil; 2 Brazil Sociedade de Cardiologia do Estado do Rio de Janeiro (SOCERJ),Rio de Janeiro, RJ - Brazil; 3 Brazil Complexo Hospitalar de Niterói (CHN), Niterói, RJ - Brazil; 4 Brazil Universidade Federal do Rio de Janeiro (UFRJ), Rio de Janeiro, RJ - Brazil; 5 Brazil Sociedade Brasileira de Cardiologia (SBC), Rio de Janeiro, RJ - Brazil; 6 Brazil Universidade do Estado do Rio de Janeiro (UERJ), Rio de Janeiro, RJ - Brazil; 7 Brazil Hospital Pró-Cardíaco, Rio de Janeiro, RJ - Brazil; 8 Brazil Pontifícia Universidade Católica de Campinas (PUC), Campinas, SP - Brazil; 9 Brazil Universidade de São Paulo (USP), São Paulo – Brazil

**Keywords:** Coronavirus Infections, Coronavirus, COVID-19, Betacoronavirus, Pandemics, Vaccination, Vaccines, Cardiovascular Diseases, Influenza, Human, Health Policy

## The COVID-19 pandemic

The World Health Organization recognized COVID-19 as a pandemic on March 11, 2020. Since then, this public health emergency has become the leading cause of death in the world and has made addressing it an unquestionable priority. At the time of writing this editorial, we counted 86 969 386 confirmed cases and 1 915 657 deaths from COVID-19 worldwide, of which, 8 013 708 cases in Brazil resulted in 201 460 deaths.^1^According to projections by the Institute for Health Metrics and Evaluation (IHME),^2^Brazil will have 248 476 deaths from COVID-19 on April 4, 2021. By March 19, 2021, 242 738 [232 202 – 255 044] deaths are estimated, and those numbers could be reduced to 241 668 [231 337 – 253 770], with the quick administration of vaccines, and to 223 910 [215 565 – 233 360], with a 95% level of face mask use in public. The magnitude of the number of cases and deaths from a single disease in such a short time is worrisome. Now, when there is a growth in the number of new cases and hospitalizations, starting to vaccinate will have an impact on reducing deaths and hospitalizations in a short interval. Despite the efforts of the scientific community, there is no specific treatment to block viral replication. In this sense, vaccination programs are powerful allies. In addition, due to the remarkable progress of science, we already have this resource.

Brazil, through its Unified Health System (SUS), has been known for the successful implementation of vaccination programs for its population. The institution of a public policy to vaccinate within the SUS principles, which are universality, integrality, and equity, is urgent. However, because of the antivaccine movements that have emerged worldwide, a strong effort to obtain the population’s adhesion is required. In the past, in the Vaccine Revolt experienced by Oswaldo Cruz, we have successfully faced that disbelief. Let us be inspired by that episode to overcome this serious health crisis.

## The epidemiology of cardiovascular diseases in COVID-19

In Brazil, between March 17 and May 22, 2020, there was a greater number of deaths in the capitals of the Northern, Northeastern and Southeastern regions, especially São Paulo, Rio de Janeiro, Fortaleza, Recife, Belém, and Manaus, and a lower number of reports of death in the capitals of the Southern and West-Central regions, and in the inner country cities. We observed an increase in the number of deaths due to non-specific cardiovascular causes in all regions, in the capitals and in the inner country, mainly in the Northern, Northeastern, and Southeastern regions. On the other hand, there was a percentage reduction in the reports of deaths from acute coronary syndrome (ACS) and stroke, with greater magnitude in the Northeastern region, followed by the West-Central and Southeastern regions (capital and inner country).^3^

In 2020, the COVID-19 pandemic in Brazil increased the number of general deaths and of deaths due to cardiovascular diseases (CVD) and non-specific causes, as well as that of sudden deaths at home. Regional differences express the socioeconomic and ethnic inequalities of a continental country, as well as the consequence of a health system with heterogeneous and poorly distributed resources.^3^

COVID-19 is the pandemic novelty and CVD are our endemic, consolidated, and irresolute reality. Both compromise health in all aspects, individually and collectively, physically, psychologically, socially, and economically. In common, they reap productive and promising lives.

We still lack double-blind, randomized, placebo-controlled studies that show the causal relationship between vaccination against COVID-19 and benefit for cardiac patients. Let us, then, use the best available evidence.

## Vaccines and the impact on humanity

Despite having arisen even before immunologists, vaccines have had an impact on the control and even eradication of once devastating diseases. Smallpox killed 29% of children in London in the 17^th^and 18^th^centuries, being declared extinct in 1980. Who among us diagnosed diphtheria myocarditis in a patient in the past ten years? How many cases of neonatal tetanus were admitted to your hospital in 2020? Vaccinations changed the natural history of some epidemics, such as that of diphtheria in 1940, of polio in 1956, of pertussis in 1950, of measles in 1968, of meningococcal disease in 1999. However, vacillation in campaigns has invariably resulted in recurrence.^4^

## The influenza model

Influenza vaccination is the successful evidence-based experience closest to the current COVID-19 pandemic situation. Although influenza vaccination is recommended by the main guidelines in cardiology, that vaccination coverage is low and has increased little in the last decade.^5^Vaccination depends to a great extent on the cardiologist’s recommendation. The cardiologist is “the clinician” of the patient with CVD, heard in several situations. The knowledge and consequent conviction about the need for a vaccine is crucial for its dissemination. The influenza vaccine is an unequivocal example: it is available, is easily accessed in campaigns, but its coverage does not exceed 25% of the patients with heart failure (HF).^5 , 6^

The need for influenza vaccination in cardiac patients has been first determined by historical reports of increased mortality in epidemics, and, later, by epidemiological studies.^5^ Table 1 shows evidence that supported these recommendations.^7 - 15^Today, influenza vaccination is known to be an effective measure for secondary prevention because it reduces hospital admissions from HF, stroke, and ACS, in addition to reducing overall mortality more significantly than many medications or interventions.^5 , 6^

**Table 1:** – Main evidence that supported the recommendation of influenza vaccination in cardiac patients

Author	Year	n	Main conclusions
Nichol KL et al.^7^	2003	286 383 elderly	Influenza vaccine reduced overall mortality by 48%, hospitalizations for heart disease by 19%, and stroke by 16-23%
Yap FHY et al.^8^	2004	17 226 admissions for NCD	Influenza caused a 45.6% increase in hospitalizations for HF
Sandoval C et al.^9^	2008	5448 patients with systolic ventricular dysfunction	The risk of hospitalization for HF is 8-10% higher during the influenza season, regardless of how it is defined
Jorge JEL et al.^10^	2009	6596 hospitalizations for HF	The seasonality with the highest number of hospitalizations for decompensated HF also occurs in tropical regions
Estabragh ZR & Mamas MA^11^	2013	40 trials	Influenza leads to direct effect: myocarditis with cardiogenic shock, increased AMI, decreased cardiovascular mortality after vaccination
Wu WC et al.^12^	2014	107 045 patients with HF	Influenza vaccination reduced mortality of patients with HF in 30 days and 1 year
Caldeira D et al.^13^	2015	4 trials	Influenza vaccination is effective in secondary prevention in patients with cardiovascular disease. Data is lacking to prove the same action in primary prevention
Blaya-Nováková V et al.^14^	2016	227 984 patients followed for 5 years	Influenza vaccination reduced risk of global winter mortality by 41% per year
Fang YA et al.^15^	2016	4406 patients with CKD and age ≥55 years.	Elderly people with chronic kidney disease who received an annual influenza vaccination have a lower risk of hospitalizations for HF

*NCD: chronic noncommunicable diseases; HF: heart failure; AMI: acute myocardial infarction; CKD: chronic kidney disease.*

## Infections and systemic inflammatory syndrome

Influenza predisposes to secondary bacterial pneumonia and, thus, decompensates patients with HF, and that is a fact. However, it should be noted that the systemic inflammatory syndrome secondary to influenza leads to changes in the coagulation factors and platelet hyperaggregability, and to an increase in inflammatory phase proteins, cytokines, and tumor necrosis factor. Consequently, there is an increase in thrombotic phenomena and fibrin deposition, cardiomyocyte hypocontractility, inflammation, acceleration of atherogenesis, and remodeling ( Figure 1 ). Thus, this easily explains the reduction in ACS and stroke in vaccinated patients as compared to controls in clinical trials and epidemiological observations.^5 , 16^

**Figure 1:** – Pathophysiology of cardiovascular changes secondary to systemic inflammation in Influenza infection. Source: The authors.

COVID-19 brought up the discussion of the same mechanisms and manifestations already well studied for influenza. It is undeniable that the COVID-19 inflammatory response is more exuberant and severe, and associated with the risk of thrombosis. Therefore, we are aware of the peculiarities of immunization in that subgroup of individuals and recommend efficient measures to increase the chances of success of the immunization program against COVID-19.

## COVID-19 and risk groups

Since the first series published from China and Italy, the severity of COVID-19 has stood out in patients with chronic noncommunicable diseases, most likely because of the chronic systemic inflammation they have in common.^17^Discounting the confusions caused by inadequate interpretations of ecological studies, the concept of risk group has remained in subsequent publications, which has been an already known fact since the studies about influenza. The patient with HF is an undoubted example of a priority group, and the Brazilian Society of Cardiology (SBC) has already expressed its opinion.^18^

Recently, the SBC has been invited by the Ministry of Health to join the Technical Chamber and review the National Immunization Program against COVID-19. The SBC has pointed out suggestions regarding vaccination in patients affected by all CVD, defining and specifying priority groups for vaccination ( Table 2 ).

**Table 2:** – Cardiovascular and cerebrovascular diseases priority to COVID-19 vaccination. Suggestions offered by the Brazilian Society of Cardiology to the National Immunization Program of the Brazilian Ministry of Health

Cardiovascular or cerebrovascular syndrome / disease	Definition
Heart failure	HF with reduced, intermediate or preserved ejection fraction, in stages B, C or D, regardless of New York Heart Association functional class Post-heart transplantation (using inactivated virus vaccines)
Cor pulmonale and pulmonary hypertension	Chronic cor pulmonale, primary or secondary pulmonary hypertension
Resistant arterial hypertension	When BP remains above the recommended targets with the use of three or more antihypertensive drugs of different classes, at maximum recommended and tolerated doses, administered frequently, appropriate dosage and proven adherence or BP controlled by using four or more antihypertensive drugs
Stage 3 hypertension	Systolic BP ≥180 mm Hg and / or diastolic BP ≥110 mm Hg regardless of the presence of TOD or comorbidity
Stage 1 and 2 hypertension with TOD and / or comorbidity	Systolic BP between 140 and 179 mm Hg and / or diastolic BP between 90 and 109 mm Hg in the presence of TOD and / or comorbidity
Hypertensive heart disease	Hypertensive heart disease (left ventricular hypertrophy or dilation, atrial and ventricular overload, diastolic and / or systolic dysfunction, injuries to other target organs)
Coronary syndromes	Chronic coronary syndromes (angina pectoris, ischemic heart disease, post-acute myocardial infarction, others)
Valvopathies	Valve lesions with hemodynamic or symptomatic repercussions or with myocardial impairment (aortic stenosis or insufficiency, mitral stenosis or insufficiency, pulmonary stenosis or insufficiency, tricuspid stenosis or insufficiency, and others)
Cardiomyopathies and pericardiopathies	Myocardiopathies of any etiology or phenotype Chronic pericarditis Rheumatic heart disease
Diseases of the aorta, large vessels, and arteriovenous fistulas	Aneurysms, dissections, hematomas of the aorta and other large vessels
Cardiac arrhythmias	Cardiac arrhythmias of clinical importance and / or associated heart disease (atrial fibrillation and flutter, and others)
Congenital heart disease in adults	Congenital heart disease with hemodynamic repercussions, hypoxemic crises, heart failure, arrhythmias, myocardial involvement
Valve prostheses and implanted cardiac devices	Individuals with biological or mechanical valve prostheses and implanted cardiac devices (pacemakers, cardioverter defibrillators, resynchronizers, medium and long-term circulatory assistance)
Cerebrovascular disease	Ischemic or hemorrhagic stroke, transient ischemic attack, vascular dementia

*HF: heart failure; BP: blood pressure; TOD: target organ damage. Source: correspondence sent to the National Immunization Program of the Brazilian Ministry of Health, on January 2, 2021.*

## The current perspectives with the different vaccines against COVID-19

There are still few vaccines tested in phase 2 or 3 studies. However, the results are positive and impactful, both in terms of safety and effectiveness. It is worth mentioning that the vaccines supported by Pfizer,^19^Moderna,^20^and AstraZeneca^21^have included the elderly, cardiac patients, diabetics, severely obese individuals, Afro-descendants, and Latinos. And, despite their relatively small number, that inclusion allows us to infer safety and efficacy for cardiac patients. The adverse effects observed were local, but less common in the elderly. The cardiovascular effects observed, such as hypertension, bradycardia, tachycardia, atrial fibrillation, ACS, and pulmonary thromboembolism, had a frequency lower than 0.1% and were similar in those who received the vaccines and those who received placebo ( Table 3 ).

**Table 3:** – Demographic and clinical characteristics of volunteers vaccinated against COVID-19 in clinical trials

Characteristics	BNT162b2 (Pfizer/BioNTech)^19^	mRNA-1273 (Moderna / NIAID / NIH)^20^	ChAdOx1 COV 003 (Oxford/ AstraZeneca)^21^
Number of volunteers (n)	44 820	30 420	23 848 (4088 Brazil)
Age range (years)	16 to 91	18 to 95	≥18
Median age (years)	52 (42.2% ≥55)	51 (24.8% ≥65)	-
Afrodescendants (%)	9	10.2	10.4**
Latin or Hispanic (%)	28	24.8	-
Adverse effects	Local pain was more common in vaccinated individuals More frequent in younger people	Local pain after injection more frequent in the vaccinated than placebo group Adverse effects are more common after the second dose Most common in younger people	not available
Cardiovascular effects	not available	Bradycardia, syncope, tachycardia, acute coronary syndrome, atrial fibrillation, hypertension, hypotension, all <0.1% and similar frequency for those receiving placebo and vaccines	not available
Efficiency (%)	95.0 [CI 90.3 – 97.6]	94.1 [CI 89.3 – 96.8]	64.2 [CI 30.7 – 81.5]
Subgroup effectiveness	Similar in subgroups, including hypertensive, elderly, obese and Brazilians	Similar in risk subgroup for severe COVID-19	not available
Obese (BMI> 30 kg/m^2^) (%)	35.1	6.7	20.4
Diabetics (%)	38.4*	9.5	3.0**
Cardiovascular disease (%)	2.7*	4.5	12.0**

BMI: body mass index. (*) Approximate calculation based on data published in the trial appendices. (**) Data refer to the series of the trial carried out in Brazil (VOC 003). Note: data obtained and analyzed with different criteria, therefore with limited comparisons.

It is worth mentioning that Brazil has entered into partnerships since May 2020 for the research and development of vaccines that include technology transfer through the Oswaldo Cruz Foundation and the Butantan Institute. The vaccine developed by AstraZeneca and the Oxford University has already had its preliminary results published, being in use in England. This vaccine will be produced on a large scale in Brazil. Concomitantly, the vaccine called CoronaVac, developed by the Sinovac laboratory, will be produced at the Butantan Institute, which has reported in the media that “ *in a clinical study with 12 400 volunteers, the immunizing agent showed 78% effectiveness for mild cases and 100% for moderate and severe cases* ”.^22^Thus, the perspective of having vaccines is good.

It is necessary to emphasize that Brazil has one of the most advanced health legislations in the world. The Brazilian Federal Constitution enshrines access to health as a fundamental right: “ *Health is a right for all and a duty of the State, guaranteed through social and economic policies* ...”. Thus, public health policies that are safe, effective, and cost-effective are part of the existential minimum of each Brazilian, and should be offered in a universal, comprehensive, and free manner. That includes the vaccination campaigns, a true consolidated patrimony of Brazilians and national pride. In view of this, creating all the conditions to offer vaccines in a comprehensive immunization program against COVID-19 is “ *a right for all and a duty of the State* ”, under penalty of constitutional duty becoming an inconsequential promise, frustrating the fair expectations deposited in the Brazilian State.

## Why vaccinate?

Figure 2 summarizes ten reasons for recommending the vaccine to your patient. It is our view, based on the best evidence available, that we should engage in the dissemination of this knowledge and motivate our patients. However, it is necessary to maintain the effective and proven measures to prevent COVID-19 spread: hand hygiene, face mask wearing, and social distancing. Even though the vaccination program may contribute to minimize spread, the classic preventive measures must certainly be maintained until the vaccination program benefit is definitively proven.

**Figure 2:** – Ten reasons to vaccinate a patient with cardiovascular disease against COVID-19. ACS: acute coronary syndrome; HF: heart failure. Source: The authors.

## The Brazilian Society of Cardiology and its commitment to science

The SBC will not escape the historical legacy, built on the example of Carlos Chagas, Dante Pazzanese, and our pioneers and transmitted for more than seven decades to more than 14 000 members, confirmed in its social purpose. The SBC’s objective is “ *to expand, disseminate and encourage, at all levels, the knowledge, diagnosis, prevention and treatment of CVD, developing educational campaigns jointly with the government and other entities and associations, and disseminating the epidemiological aspects of CVD to the civil society, which should be educated about the prevention and treatment possibilities* ”.^23^

Despite the high cost of lost lives, the search for an efficient solution to the pandemic has brought us rapid advances in research, based on good quality science, leaving a remarkable legacy and achievements. In one year, the clinical picture, the epidemiological profile and the etiological agent at the molecular level were described, care was improved, empirical and futile treatments were refuted, and vaccines tested in clinical trials were produced. This is science in its fascinating evolution for effectiveness in favor of quality and quantity of life. However, the great lesson has been the need to strengthen the health system, our SUS.

The uncompromising defense of SUS, in short, is the defense of the dignity of the human person, a fundamental commitment of the Brazilian State. The SBC and the other scientific societies must ally themselves in the fight for the progress and diffusion of science and for the achievement of public policies capable of improving the lives of each of the more than 220 million Brazilians. The principles that guided the creation of the SBC in 1943, in the middle of the Second World War, are still the same that motivate us in this unprecedented health crisis.
